# Alois Alzheimer (1864-1915): The Father of Modern Dementia Research and the Discovery of Alzheimer’s Disease

**DOI:** 10.7759/cureus.71731

**Published:** 2024-10-17

**Authors:** Vishwa S Thakor, Anisha Tyagi, James M Lee Jr., Frederick Coffman, Rahul Mittal

**Affiliations:** 1 Health Informatics, Rutgers University, Piscataway, USA; 2 Orthopedic Surgery, Rutgers Robert Wood Johnson Medical School, New Brunswick, USA; 3 Orthopedic Surgery, Orange Orthopedic Associates, West Orange, USA

**Keywords:** biographies, historical vignette, historical vignettes, medical innovation, medical stories

## Abstract

Alois Alzheimer was a German psychologist and neuropathologist who significantly advanced the study of dementia with his discovery of Alzheimer’s disease (AD). Based on his assessment of a 51-year-old female patient with symptoms of presenile dementia and after conducting a postmortem autopsy of her brain, Alzheimer distinguished two neurological substances - senile plaques and neurofibrillary tangles - as unique markers of what was later deemed as AD. He recognized that dementia is not a natural consequence of age but rather a recognizable neurocognitive disorder. Despite the long-lasting criticism of his findings, Alzheimer’s discovery fundamentally altered the landscape of neuropathological studies by establishing that AD was a clinically identifiable disease with distinct markers that could be targeted for treatment. Today, modern research on AD continues to build on the foundation laid by Alzheimer’s discovery.

## Introduction and background

**Figure 1:**
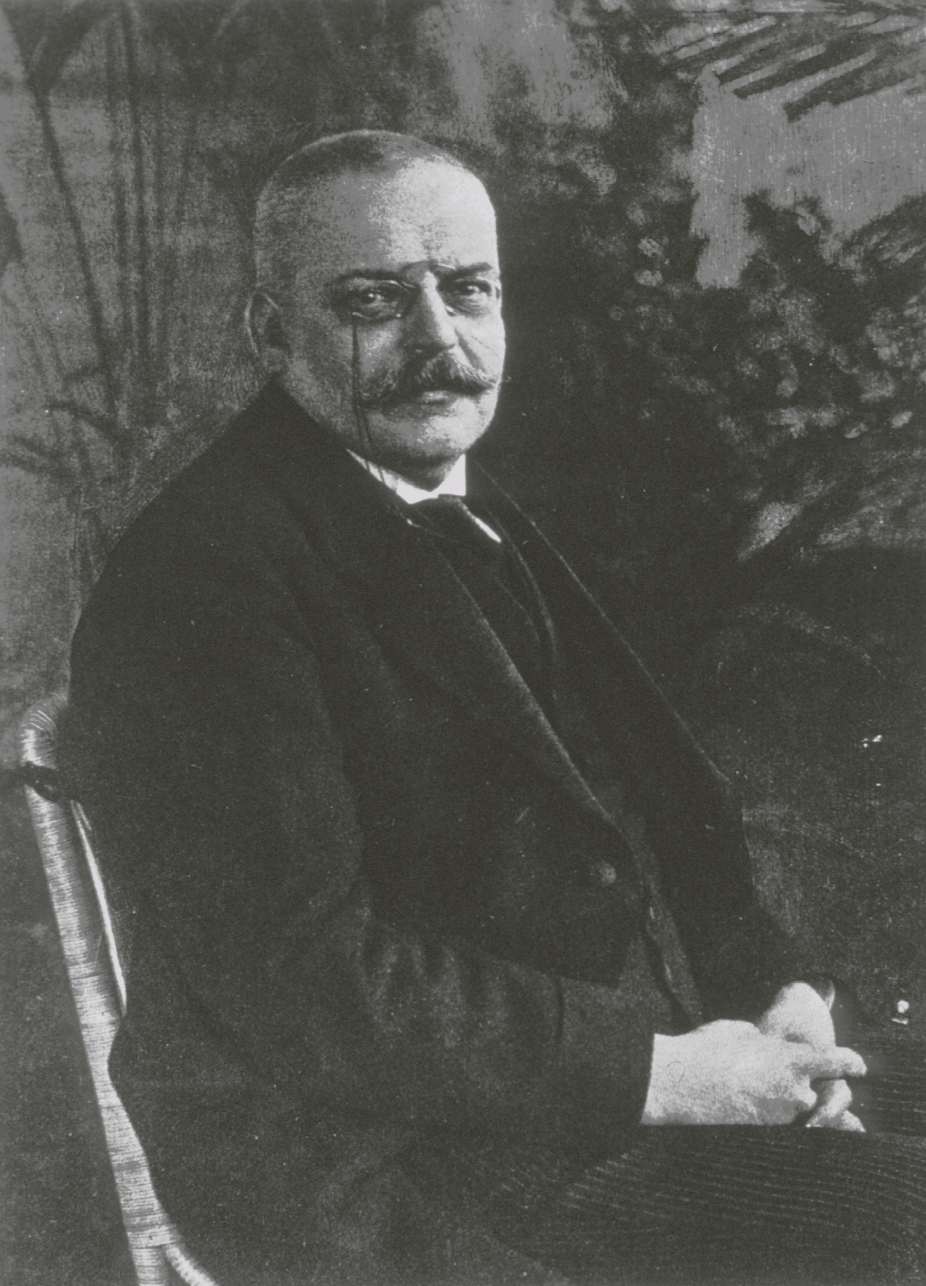
Alzheimer, Alois, 1864-1915 Credit: National Library of Medicine, Public Domain, via National Library of Medicine Digital Collections [5]

This article explores the remarkable work of Alois Alzheimer (Figure 1) in the field of psychiatry during a time when significant stigma was associated with mental illnesses and relatively few medical professionals researched the biological basis of mental disorders. Alzheimer, a German psychologist and neuropathologist, encountered a case of what he described as “an unusual disease of the cerebral cortex” with associated neurological histology, and this discovery went down in the books as Alzheimer’s Disease [1-4]. Alzheimer’s discovery greatly expanded scientific knowledge of dementia by establishing a strong correlation between altered brain anatomy and abnormal brain function, counteracting the belief that the disease was simply age-related [2,5].

**Figure 2:**
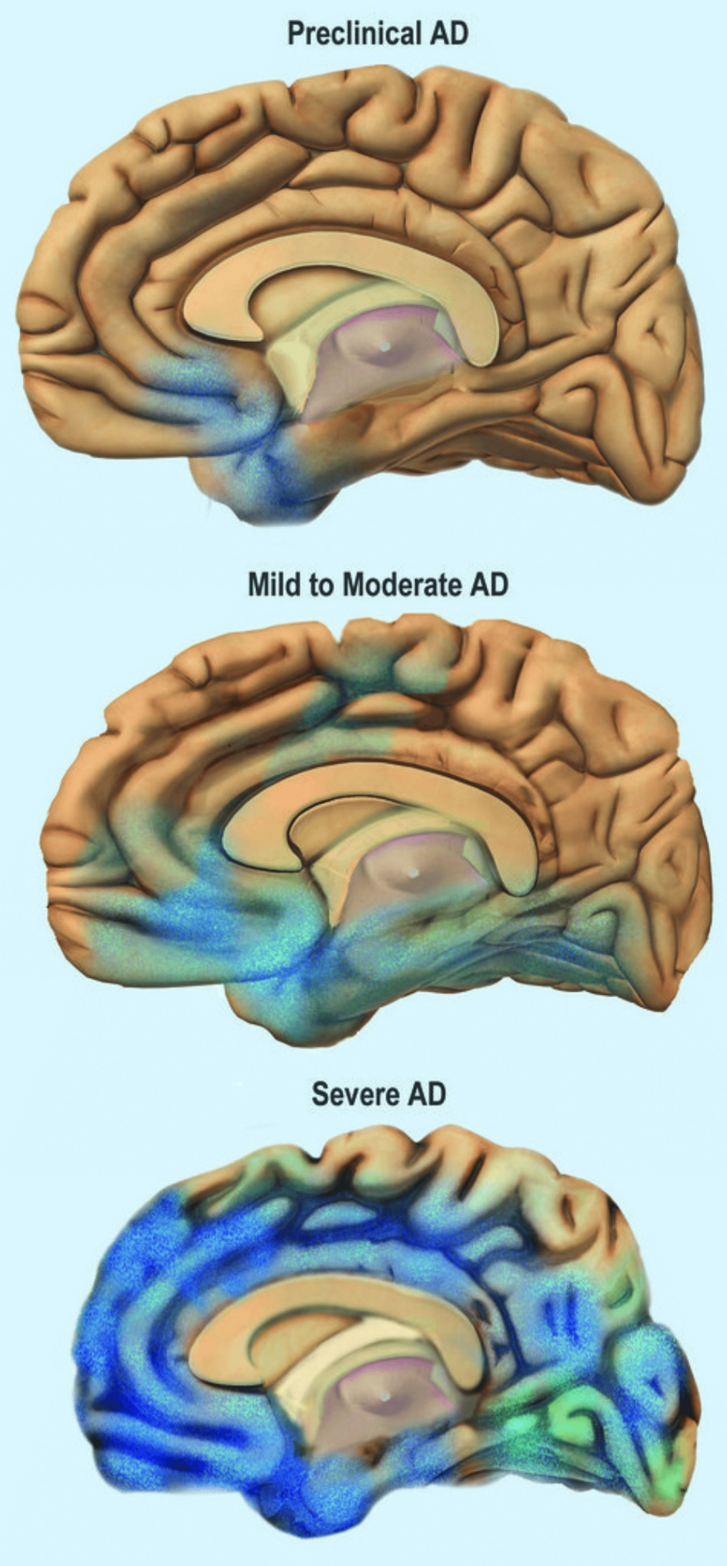
Progression of brain deterioration and loss of brain tissue in Alzheimer’s disease Credit: National Institute on Aging and National Institutes of Health, Public Domain, via Flickr NIH Image Gallery [7] AD: Alzheimer’s disease

Alzheimer’s Disease (AD) is a form of dementia characterized by memory loss, delirium, and behavioral regression, paired with significant brain tissue atrophy (Figure 2) and abnormal histopathologic changes [6-9]. During Alzheimer’s early life, the condition of dementia (more specifically, senile dementia) was considered a natural part of aging, and little was known about the pathology of the disorder. Alzheimer’s research played a crucial role in advancing psychiatric sciences by challenging older claims about senile dementia with newer research examining the histologic pathology of presenile dementia. Though Alzheimer did not find a pathological cause of AD, he identified two neurological markers of the condition -neurological tangles and senile plaques -demonstrating that AD was an illness and not a natural life process, thereby opening avenues for clinical treatment [3,6].

## Review

Alzheimer’s education and career

The son of Eduard Alzheimer and his second wife Theresia Busch, Alois Alzheimer was born in 1864 in the small town of Marktbreit-am-Main, Germany [4,10]. Alois Alzheimer’s academic journey began at the Catholic Primary School in Markbreit and progressed onto the Royal Humanistic Secondary School in Ascheffenburg where he graduated in 1883. He then began his collegiate studies at Fredrich-Wilhelm University in Berlin, transferred to the Julius-Maximilian University in Würzburg in 1884, and to the Eberhard Karls University in Tübingen in 1886, finally graduating from Würzburg, as a doctor of medicine with the highest honors [2]. During his time at Julius-Maximilian, Alzheimer published his first thesis under anatomist and physiologist R. Von Kolliker (1817-1905) regarding the ceruminous glands of the ear, marking his entry into the realm of research [4-5,11].

Shortly after his time with Kolliker, Alzheimer agreed to serve as the personal physician for a mentally ill woman for five months at sea, an experience which many have thought to be the initial spark for Alzheimer’s lifelong pursuit of psychiatry and neuropathological research. In 1888, Alzheimer joined the Municipal Mental Asylum in Frankfurt-am-Main as an assistant clinician, where he met two of his most influential mentors, Dr. Emil Sioli (1852-1922) and Franz Nissl (1860-1919), and pursued much of the early research into AD. In 1892, Alzheimer began caring for Nathalie Geisenheimer, the widow of a wealthy diamond merchant who used her wealth to support the young doctor, enabling Alzheimer to fund many of his laboratory and research projects independently. In April of 1894, Alzheimer married Cecilie Simonette, with whom he had three children. Sadly, Cecilie passed away in early 1901, and Alzheimer, choosing not to remarry, dedicated himself entirely to his work for the remainder of his life [3,4,11].

In 1903, Alzheimer transferred to the Heidelberg Psychiatric Clinic in Munich under Emil Kraeplin (1856-1926), who would later be an invaluable asset to Alzheimer’s career. Alzheimer was soon hired as the head of the Munich Institute of Psychiatry laboratory, where he received the resources and opportunities to study dementia in depth. In 1912, Alzheimer pursued the last years of his career as the Chair of Psychiatry and Neurology for the University of Breslau, mentoring students and contributing to medical journals. Alzheimer passed away of cardiac failure and uremia on December 19, 1915, at the age of 51, largely unaware of the milestone he left behind for psychiatry and neuropathology [2-5].

Notable influences

Alzheimer did not have the family guidance or influence many figures of similar stature had in their respective fields. Hence, his true advancements and fulfillment came from his time with his mentors, or rather, his friends. Dr. Emil Sioli, a psychiatrist at Frankfurt Hospital, was the first to make a valuable impact on Alzheimer’s career. Dr. Sioli, with his insights into neuropsychiatric pathology, is credited with sparking Alzheimer’s early interest in cognitive decline and neurological disorders. Dr. Sioli was also the first to formally teach Alzheimer neuropsychiatric pathology, which turned out to be crucial in Alzheimer’s later research in the organic origins of dementia [2,4].

The most valuable influence on Alzheimer’s life was Franz Nissl, a colleague who joined Frankfurt Hospital under Dr. Sioli one year after Alzheimer’s placement. Nissl was not only Alzheimer’s coworker in research and one of Alzheimer’s dearest friends, but he was also an important figure in the early development of neuropathology himself. Nissl is credited with developing a staining method that allows granular bodies containing ribosomes and endoplasmic reticulum (called Nissl bodies or Nissl substances) to be seen within neurons. Changes in Nissl bodies have proven to be important biomarkers of neuronal injury, because the dispersal of Nissl bodies to the periphery of neurons is a sign of reversible hypoxic injury, while the disappearance of Nissl bodies is a hallmark of irreversible neuronal injury. With his experience in these microscopic studies, Nissl taught Alzheimer various histopathological techniques that could be applied to microscopic observations and kindled Alzheimer’s interest in microscopic research [2,4,10-12].

The biggest asset to Alzheimer’s professional growth was psychiatrist Emil Kraeplin. Kraeplin guided Alzheimer’s interest toward the histopathological origins of mental illnesses, which was pivotal in sparking Alzheimer’s research on AD. Later, Kraeplin was responsible for bringing Alzheimer’s claims and findings to light and terming the newly discovered form of dementia as Alzheimer’s Disease [4,6,10-11,13].

The discovery of Alzheimer’s disease

**Figure 3:**
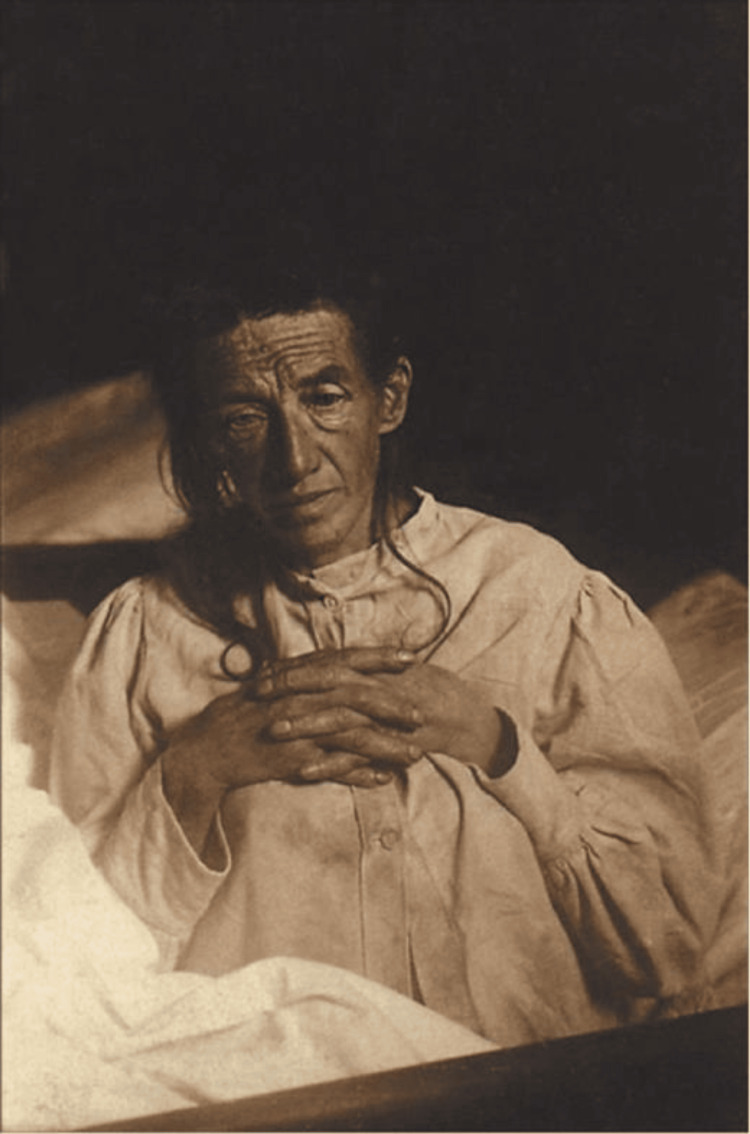
Deter, Auguste, 1850-1906 Credit: Store Norske Leksikon (Large Norwegian Encyclopedia), Public Domain [14]

In 1901, Alzheimer encountered a 51-year-old female patient, Auguste Deter (1850-1906) (Figure 3), who was admitted to Frankfurt Hospital for paranoia, insomnia, disorientation, memory loss, and aggression. Alzheimer studied and interacted with Auguste throughout his time in Frankfurt, and though Alzheimer transferred to Munich in 1903, he kept up with the documented progression of Auguste’s condition throughout the following years [2,4].

Auguste initially only showed the symptoms reported by her husband upon admittance but later presented delusions, auditory hallucinations, fear of nonexistent situations and entities, helplessness, screaming, crying, and expanded disorientation and confusion as well. Several tests revealed that Auguste had a perception disorder with objects, experienced short-term memory loss, omitted sentences and syllables when reading, speaking, and writing, and experienced moments of absent-mindedness and prolonged confusion, all while having completely normal physical reflexes. Auguste’s mental regression continued steadily and she was reported completely apathetic by the time of her death in 1906 [4,10,12].

**Figure 4:**
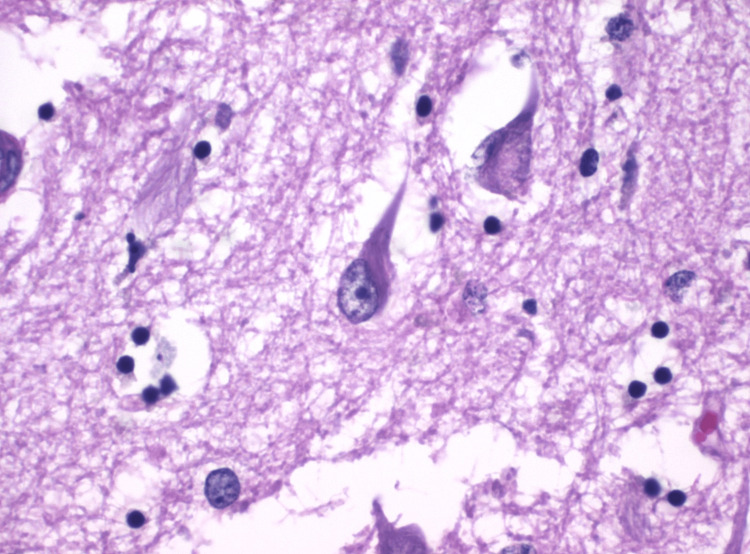
Presence of neurofibrillary tangles (dark purple streaks) in the hippocampus of the brain exhibiting Alzheimer’s disease Credit: Mikael Häggström, Public Domain, via Wikimedia Commons [15]

Alzheimer kept up with all of this data and oversaw an autopsy performed on Auguste’s brain within 20 days of her death. The autopsy revealed cortical atrophy, minor cerebrovascular changes, thickened neurofibrils that led to neurofibrillary tangles, and the presence of senile plaques (Figure 4) in Auguste’s brain. The most significant finding of the autopsy was the presence of neurofibrillary tangles and senile plaques, which Alzheimer recognized as having never been seen before and being unique to only this neurological condition. Upon further study, Alzheimer was not able to find a pathological cause of the disease but concluded that the senile plaques were associated with central nervous system degeneration and were markers of this form of dementia. Alzheimer’s discovery also indicated that senile dementia was not an event correlated only with the natural progression of age, but rather due to identifiable pathology, opening avenues for clinical treatments and therapies to prevent or reduce the progression of dementia [3-6,10,12].

Response of the scientific community

In November of 1906, Alzheimer presented his newly discovered form of dementia at the 37th Assembly of Southwest German Psychiatrists in Tübingen, Germany. The response to Alzheimer’s discovery by the psychiatric population was rather disappointing, and the chairman of the assembly, Alfred Hoche (1865-1943), took little interest in commenting on Alzheimer’s lecture or urging the assembly to discuss his claims. Only a two-line abstract of the Alzheimer’s lecture was found in the proceedings of the assembly [2,11-12].

However, in 1910, the minor but significant change that gave Alzheimer his place in the history books occurred - Kraeplin’s publishing of Alzheimer’s research in the textbook Psychiatrie A, regarding the histological changes occurring in presenile dementia, where Kraeplin coined the term “Alzheimer’s disease” that we all know today [1-3]. Regardless of the official publication, the scientific society of the time still did not appreciate Alzheimer’s findings, and his work was deemed inappropriate for the counterclaims it made against dementia being a natural process of aging [3-4,6,13].

Alzheimer’s discovery remained buried for nearly half a century before it resurfaced in the 1980s. American scientists George Glenner (1927-1995) and Cai’ne Wong took notice of Alzheimer’s work in 1984 and studied senile plaques further, identifying beta-amyloid proteins (the substance that the senile plaques are composed of) as playing a prime role in the cell regression of AD, which proved to be an incredibly significant finding in the development of modern pharmaceutical treatments [16-17]. Although recent studies have introduced additional biological factors into the increasingly complex set of mechanisms involved in AD pathology (such as inflammation, ApoE variants, and interactions with the gut microbiome), Alzheimer’s work was invaluable in defining this form of senile dementia as a medical and psychological disorder and giving rise to the plethora of studies and treatments that exist today [10,12-13,17].

## Conclusions

Alois Alzheimer made the profoundly crucial discovery of a specific form of dementia and the neurological markers associated with it. Today, AD is a widely recognized condition present in a significant segment of the older population and Alzheimer’s discovery has led to many treatments that delay the onset of symptoms and improve the quality of life for those affected. Alzheimer’s work was a landmark in psychiatric practice and neuropathological studies, greatly expanding our understanding of the presentation of neurological illnesses.
